# Serum folate levels and hypertension

**DOI:** 10.1038/s41598-022-13978-5

**Published:** 2022-06-16

**Authors:** Yoonkyung Lee, Sangshin Park

**Affiliations:** 1 Graduate School of Urban Public Health, University of Seoul, 163 Seoulsiripdae-ro, Dongdaemun-gu, Seoul, 02504 Republic of Korea; 2 Department of Urban Big Data Convergence, University of Seoul, Seoul, Republic of Korea

**Keywords:** Diseases, Medical research, Risk factors

## Abstract

We aimed to examine the association between serum folate levels and hypertension in Korean adults. Our study population was 6343 Korean adults whose blood pressure and folate levels were measured in the Korea National Health and Nutrition Examination Survey 2016–2018. We aggregated the study populations into quintiles according to serum folate levels (1.5–4.3, 4.4–5.7, 5.8–7.5, 7.6–10.3, and 10.4–35.9 ng/mL). Multivariable logistic and linear regression models were used to analyze the relationships between serum folate levels, blood pressure, and hypertension. The weighted average of serum folate levels was 7.4 ng/mL, and the weighted prevalence of hypertension was 30.4% in the study populations. After adjusting for all potential confounders, compared to those in the lowest quintile of serum folate levels, systolic and diastolic blood pressure of the people in the other quintiles were not significantly different. The linear relationship between serum folate levels and blood pressure was not statistically significant. The odds for hypertension were not significantly different across the quintiles of serum folate levels. This study showed high serum folate levels was not significantly associated with lowering hypertension in Korean adults.

## Introduction

Hypertension is characterized by continuous elevation in arterial pressure. It increases the risk of stroke^1^, heart disease^2^, kidney failure^3^, disability^4^, and premature mortality^5^. Abnormal blood pressure (BP) has been linked to nearly 50% of all cases of ischemic heart disease and two-thirds of stroke incidence^6^. Globally, 64.3 million disability-adjusted life years accounting for 4.4% of the total incidence worldwide and 7.1 million deaths constituting 12.8% of the global incidence were presumed to be caused by abnormal BP^6^. Therefore, determining the potential risk or preventive factors for hypertension is crucial for public health.

As a water-soluble B vitamin, folate is naturally present in a wide variety of foods, containing vegetables (e.g., asparagus, spinach, and brussels sprouts), fruits, beans, nuts, peas, eggs, seafood, dairy products, grains, and meat^7^. It is also important for conversion of homocysteine to methionine^7^. In the absence of adequate folate supplement, the homocysteine levels can be raised. High concentrations of homocysteine are associated with elevated BP^8^. Therefore, the Korea Ministry of Health and Welfare and the Korean Nutrition Society defined the recommended intake level of folate^9^. The recommended folate intake for Korean adults is 400 μg dietary folate equivalent (DFE)/day, and the tolerable upper intake level of folic acid in adults is 1000 μg/day regardless of gender or age^9^. The average daily intake of folic acid for Korean adult men was 352.0 μgDFE and for women it was 288.4 μgDFE^10^. Korean aged 50 to 64 years had the highest folic acid intake for both men, 394.9 μgDFE, and women, 332.5 μgDFE^10^.

Nevertheless, only few studies showed that folate intake has a positive effect on reducing the risk of hypertension or high BP by lowering the concentration of homocysteine^11–13^. These studies have two limitations. First, previous studies used a questionnaire-based folate intake estimates as an independent variable^11,13^, instead of serum folate levels, which is a more direct indicator of folate status. Second, these studies were performed on preschool children^13^ or young adults (18–30 years old)^12^. Therefore, the aim of this study was to examine the direct relationship between serum folate levels and hypertension using a representative Korean adult data.

## Materials/subjects and methods

### Study population

We analyzed the data from the Korea National Health and Nutrition Examination Survey (KNHANES) 2016–2018 (http://knhanes.cdc.go.kr/) which is a cross-sectional survey to investigate health and nutritional status of Korean. To collect nationally representative population, Korea Centers for Disease Control and Prevention (KCDC) utilizes stratified and multistage probability cluster sampling method. KCDC collects in individual data through household member confirmation survey, health survey, health examination survey, and nutrition survey. All participants signed written consent forms to participate to the KNHANES. The KNHANES were conducted in accordance with the ethical principles of the Declaration of Helsinki. The KNHANES data were anonymized prior to release to the public. The KNHANES is described in detail elsewhere^14^.

To address our research question, out of 19,197 participants ≥ 20 years in the KNHANES 2016–2018, we analyzed the data of 6343 participants who were measured for serum folate levels and BP. Our study protocol was exempted from the ethical review by the Institutional Review Board of the University of Seoul (UOS-IRB-2020-27).

### Primary predictor and outcome of interest

Hypertension was defined as either systolic BP (SBP) ≥ 140 mmHg or diastolic BP (DBP) ≥ 90 mmHg, physician-diagnosed hypertension, or taking antihypertensive medicine. The nurse measured BP three times using a standard mercury sphygmomanometer [Baumanometer Wall Unit 33(0850); Baum Co., Inc., Copiague, NY]. We used the average of secondary and tertiary BP. The serum folate levels were measured by the Chemiluminescent Microparticle Immunoassay (ARCHITECT i4000Sr; Abbott, Wiesbaden, Germany).

### Covariates

The KNHANES used face-to-face interviews to collect the information on age, sex, household income, education, occupational classification, and marital status. We divided participants into four groups according to the monthly household income; two groups according to the level of education (≤ middle school or ≥ high school); three groups according to occupation (white collar/service sales, blue collar, or unemployed categories); and two groups according to marital status (married or unmarried/bereaved/separated/divorced).

The KNHANES used self-administered questionnaire to collect the information on smoking status, high-risk drinking, and strength exercise. We divided participants into two groups according to smoking status (current smoking ≥ 100 cigarettes in a lifetime or not); two groups according to drinking status (high-risk drinking ≥ 2 times/week and ≥ 7 cups/time in men and ≥ 5 cups/time in women or not); and two groups according to strength exercise status (≥ 2 days/week or not). The KNHANES performed 24-h recall survey to collect the nutrient intakes information, such as daily energy and sodium intakes.

We also used the data of body mass index (BMI), diabetes mellitus, and family history of hypertension in health examination survey of the KNHANES. The height was measured by the coordinator using a stadiometer (SECA 225; Vogel & Halke, Hamburg, Germany), and the weight was measured using a scale (GL-6000-20; CAS Korea, Seoul, South Korea). BMI was calculated by dividing weight in kilograms by squared height in meters. Obesity was defined by a BMI ≥ 25 kg/m^2^^15^. Blood glucose levels were measured using an autoanalyzer (model 7600 automatic analyzer; Hitachi, Tokyo, Japan). Diabetes mellitus was defined by ≥ 126 mg/dL of fasting blood glucose level, a diagnosis by a physician, or ongoing treatment with an antidiabetic medication or insulin injections.

### Statistical analysis

We aggregated participants into quintiles according to serum folate levels (1.5–4.3, 4.4–5.7, 5.8–7.5, 7.6–10.3, and 10.4–35.9 ng/mL). To examine the relationship between serum folate levels and BPs and hypertension, we performed multivariable linear and logistic regression analysis with adjustments for age, sex, marital status, education level, household income, occupational status, smoking status, high-risk drinking, strength exercise, daily energy intake, daily sodium intake, family history of hypertension, obesity, and diabetes mellitus. We tested whether sex play a role as an effect modifier in the relationship between serum folate levels and BP or hypertension in the multivariable models. However, because the effect modification of sex was not found in each model, we performed statistical analyses at once regardless of sex. All statistical analyses were conducted using SAS 9.4 version (statistical analysis system; SAS Institute Inc., Cary, NC). The *p* value was considered statistically significant when it was less than 0.05.

## Results

The average age of participants with hypertension in the study was 57.3 years, which was significantly higher than the age of those without hypertension, 41.9 years (Table 1). Serum folate levels in participants with hypertension were not significantly different from those without hypertension. Participants with hypertension were more likely to have obesity, diabetes mellitus, and have family history of hypertension; they are more likely to smoke and engage in high-risk alcohol drink.

**Table 1:** Characteristics of study participants.

Variable	Without hypertension		With hypertension		*p*
	Mean or proportion	SE	Mean or proportion	SE	
N (%)	4416 (69.6)		1927 (30.4)		
Female, %	57.8		48.7		
Age, y	41.9	0.2	57.3	0.4	< 0.001
Education level: ≥ high school, %	86.4	0.6	59.6	1.5	< 0.001
Household income, %					
Quartile 1 (Lowest)	11.0	0.6	24.3	1.2	< 0.001
Quartile 2	23.7	0.9	24.3	1.2	
Quartile 3	32.3	0.9	24.7	1.2	
Quartile 4 (Highest)	33.0	1.1	26.7	1.4	
Occupation, %					
White collar/Service sales	47.6	1.0	33.9	1.3	< 0.001
Blue collar	20.6	0.8	27.4	1.3	
Unemployed	31.8	0.9	38.7	1.4	
Marital status: single, %	36.2	0.9	28.2	1.3	< 0.001
Cigarette smoking, %	39.5	0.9	48.2	1.4	< 0.001
High-risk alcohol drink, %	12.9	0.6	17.5	1.1	< 0.001
Strength exercise, %	23.2	0.8	20.5	1.2	0.08
Obesity, %	29.0	0.8	50.6	1.4	< 0.001
Diabetes mellitus, %	4.9	0.5	24.6	1.7	< 0.001
Family history of hypertension, %	40.0	0.9	55.1	1.5	< 0.001
Energy intake,^a^ kcal/d	1908	29.1	1882	42.7	0.90
Sodium intake,^a^ mg/d	3123	67.4	2968.1	181	0.80
Serum folate levels,^a^ ng/mL	6.4	0.2	6.7	0.2	0.11

^a^Data present median and SE.

Without adjustments, DBP of participants with 10.4–35.9 ng/mL serum folate levels were significantly different from that of those with 1.5–4.3 ng/mL and 5.8–7.5 ng/mL; a significant linear trend was observed across quintiles (p for trend: 0.046) (Table 2). However, after adjusting for all covariates, SBP and DBP were not significantly different across quintiles of serum folate levels; significant linear trends were not observed.

**Table 2:** Least square means (SE) of SBP and DBP according to quintiles of serum folate levels.

	Quintile of serum folate levels (range, ng/mL)					*p* for trend
	Q1 (1.5–4.3)	Q2 (4.4–5.7)	Q3 (5.8–7.5)	Q4 (7.6–10.3)	Q5 (10.4–35.9)	
N	1277	1228	1289	1289	1260	
**SBP**						
Model 1	116.4 (0.9)	116.8 (1.0)	117.4 (1.2)	116.6 (0.9)	116.2 (1.0)	0.88
Model 2	118.6 (0.9)	118.2 (1.0)	118.0 (1.1)	117.3 (0.9)	116.1 (0.9)	0.07
Model 3	118.1 (0.9)	118.0 (1.0)	117.5 (1.4)	116.8 (1.0)	116.9 (1.0)	0.25
**DBP**						
Model 1	76.7 (0.7)^a^	76.4 (0.7)	77.1 (0.6)^b^	76.0 (0.5)	74.8 (0.6)^a,b^	0.046
Model 2	75.6 (0.7)	75.8 (0.7)	76.8 (0.6)	76.5 (0.5)	75.4 (0.6)	0.83
Model 3	75.9 (0.7)	75.7 (0.7)	76.2 (0.6)	76.5 (0.7)	75.7 (0.6)	0.91

Model 1: not adjusted; model 2: adjusted for age and sex; model 3: adjusted for age, sex, education level, household income, occupation, marital status, cigarette smoking, high-risk drinking, strength exercise, obesity, diabetes mellitus, family history of hypertension, energy intake, and sodium intake. Mean in a row with common superscript indicates significant difference, p < 0.05.

We found the significant relationship between the continuous predictor, serum folate level, and DBP (*B*: − 1.406, p: 0.011) without adjustments; SBP (*B*: − 1.618, p: 0.046) with adjustments for age and sex (Table 3). However, after adjusting for all covariates, these significant relationships disappeared. With or without adjustments, the continuous predictor, serum folate level, was not significantly associated with hypertension (Table 4).

**Table 3:** Associations of serum folate levels with SBP and DBP.

	*B* (95% CI)	β	*p*
**SBP**			
Model 1	− 0.106 (− 1.685, 1.473)	− 0.003	0.90
Model 2	− 1.618 (− 3.209, − 0.027)	− 0.053	0.046
Model 3	− 1.140 (− 2.733, 0.453)	− 0.038	0.16
**DBP**			
Model 1	− 1.406 (− 2.480, − 0.333)	− 0.072	0.011
Model 2	− 0.136 (− 1.239, 0.968)	− 0.007	0.81
Model 3	− 0.197 (− 1.314, 0.920)	− 0.010	0.73

*B*: unstandardized coefficient; β: standardized coefficient; model 1: not adjusted; model 2: adjusted for age and sex; model 3: adjusted for age, sex, education level, household income, occupation, marital status, cigarette smoking, high-risk drinking, strength exercise, obesity, diabetes mellitus, family history of hypertension, energy intake, and sodium intake. Serum folate levels (ng/mL) were naturally log-transformed.

**Table 4:** The association between serum folate levels and hypertension.

	OR (95% CI)	*p*
Model 1	1.203 (0.962, 1.505)	0.11
Model 2	0.907 (0.683, 1.206)	0.50
Model 3	0.885 (0.615, 1.274)	0.51

Model 1: not adjusted; model 2: adjusted for age and sex; model 3: adjusted for age, sex, education level, household income, occupation, marital status, cigarette smoking, high-risk drinking, strength exercise, obesity, diabetes mellitus, family history of hypertension, energy intake, and sodium intake.

## Discussion

This study showed that serum folate levels were not significantly associated with SBP, DBP, or odds of hypertension in Korean adults. Contrary to our results, several epidemiologic studies have showed that higher folate intakes were associated with decreased levels of BP^13^ and a decreased risk of hypertension^11,12^.

For example, Tamai et al. reported that children aged 3–6 years in the highest quintile (≥ 229 μg/d) of folic acid intake had a SBP of 4.1 mmHg lower than did those in the lowest quintile (≤ 156 μg/d)^13^. Forman et al. reported that women who consumed ≥ 1000 μg/d of folate had decreased risks of hypertension than those who consumed < 200 μg/d in the Nurses’ Health Study cohort [aged 27–44 years at baseline, relative risk (RR) = 0.54, 95% CI 0.45–0.66; aged 43–70 years at baseline, RR = 0.82, 95% CI 0.69–0.97]^11^. Xun et al. also reported that high folate intakes in men and women aged 18–30 years were associated with a decreased incidence of hypertension later in life^12^. Moreover, a meta-analysis study on 30 randomized controlled trials found 4% decreased risks for cardiovascular diseases (CVDs) and 10% decreased risks for stroke in participants receiving folic acid supplementation^16^.

Several mechanisms in the relationship between lack of folate intake and elevated BP may explain the previous studies’ findings. First, low- or sub-normal levels of serum folate appear to induce an accumulation of homocysteine^17^. This homocysteine leads to homocysteine-induced arteriolar constriction, increased sodium reabsorption, renal dysfunction, and increased arterial stiffness^18^, which ultimately results in increased BP. Second, 5-methyltetrahydrofolate, an active form of folate in vessel, increases endothelial function by enhancing the activity of nitric oxide in endothelial cells of blood vessels. Thus, inadequate levels of serum folate can increase BP through endothelial dysfunction^19^.

Nevertheless, unexpectedly, our study found that serum folate levels were not significantly associated with SBP, DBP, or odds of hypertension. It is difficult to explain the mechanism by which BP was not affected by serum folate levels, but several possibilities for these results can be proposed. Some nutrients correlated with folate intake might have played a role in lowering BP in the previous studies^11–13^. Alternatively, genetic or age differences between study population might have influenced the inconsistent relationship between serum folate levels and BP or odds of hypertension. Since the effect of folic acid on vasodilation has already been well established^20^, future studies are needed to elucidate the causes of the differences in the folate-BP relationship between our and previous studies’ findings.

Our study has some strengths. This study examined the relationship between folate and BP and odds of hypertension more accurately by using serum folate levels instead of dietary folate intake. Moreover, we used large-scale national representative data to study our research question. However, because this study is a cross-sectional study, there was a limitation in that causality could not be inferred. This study has another limitation in not being able to approach the relationship between long-term folate levels (e.g., red blood cell folate^21^) and BP. Alternatively, we used serum folate levels which are indicative of recent folate intakes.

In conclusion, this study demonstrated that serum folate levels were not significantly associated with BP or odds of hypertension in Korean adults. This finding indicates that dietary folate intake or folate supplementation is unlikely to help lower the risk of hypertension. Considering that our study results are different from previous studies, further epidemiologic studies are needed to determine the detailed effects of folate on BP.

## Data Availability

The KNHANES data is publicly available (http://knhanes.cdc.go.kr). No permission is required to access the data.
